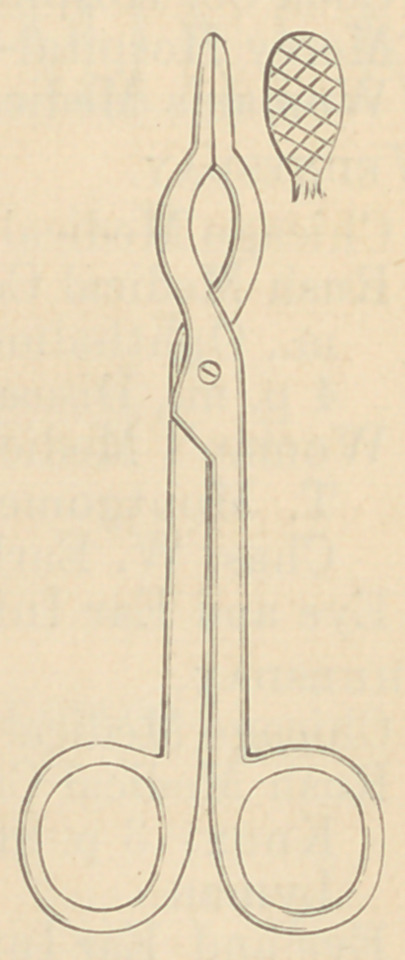# Items

**Published:** 1883-02

**Authors:** 


DEFLECTED SEPTUM NARIUM. — For the relief of this unpleasant deformity, after trying various other methods, I have lately practiced the following operation :

I make a crucial incision through the septum, one line of which is longitudinal, the other transverse. These incisions are made with the utmost obliquity practicable, so that the four quadrants imbricate upon one another, thus permitting them to easily override one another when the deflection is rectifiod.

The rectification may be effected with a pair of ordinary polypus forceps ; but I have had constructed for this purpose a pair after the accompanying sketch.

After rectification, I insert into the heretofore occluded nostril a rubber tube of the required size, which is worn for two or three weeks.

MOSES GUNN.

TO MEDICAL SOCIETIES.—Secretaries of Medical Societies in the State of Illinois are requested to forward lists of the officers and members of such societies, with post-office addresses, to the Secretary of the State Board Health at Sprinfield. These lists are needed to facilitate the distribution of the publications of the Board and for other purposes.

CHICAGO MEDICAL COLLEGES.—The winter sessions of the various medical colleges will soon close. They have all had a winter of great prosperity, and the large classes demonstrate the importance of Chicago as a medical center.

## Figures and Tables

**Figure f1:**